# Determinants of medical students’ attitudes toward artificial intelligence: a cross-sectional study and implications for medical education

**DOI:** 10.1186/s12909-026-09859-4

**Published:** 2026-07-08

**Authors:** Batuhan Horasan, Ahmet Ergin, Eda Şenarabacı

**Affiliations:** 1 Denizli, Türkiye; 2 Department of Public Health, Faculty of Medicine, Pamukkale University, Denizli, Türkiye; 3 Faculty of Medicine, Pamukkale University, Denizli, Türkiye

**Keywords:** Artificial intelligence, Attitude, Medical students, Medical education

## Abstract

**Background:**

Artificial intelligence (AI) has emerged as one of the most rapidly evolving technologies in recent years and is increasingly being integrated into healthcare, education, and everyday life. Examining university students’ attitudes toward this technology is important for understanding their future professional orientations and adaptation to technological change. This study aimed to identify medical students’ attitudes toward artificial intelligence and the determinants of these attitudes, and to develop educational implications for medical training based on the findings.

**Methodology:**

This descriptive cross-sectional study was conducted between January 22 and May 28, 2025, among 198 final-year medical students at Pamukkale University Faculty of Medicine in Denizli, Türkiye. Data were collected using a Descriptive Information Form, which assessed students’ sociodemographic characteristics and their knowledge and experiences regarding AI, and the General Attitudes toward Artificial Intelligence Scale (GAAIS, Turkish version). Since the negative subscale is reverse-coded, higher scores in both indicate more positive attitudes toward AI. Data were analyzed using SPSS v25 with descriptive statistics, Mann-Whitney U, Kruskal-Wallis, and multiple linear regression analyses.

**Results:**

The mean age of participants was 24.47 ± 0.98 years; 56.1% were female. A total of 74.2% of the participants reported general knowledge about AI, and 76.3% reported experience using AI in daily life. A total of 63.1% viewed AI developments positively, 69.7% believed AI changes work and daily life, and 52.0% felt emotionally unaffected by it. The mean positive attitude score was 45.01 ± 9.17, and the negative attitude score was 26.41 ± 6.22. Multiple linear regression analysis showed that positive attitudes toward artificial intelligence were significantly associated with father’s education level (university vs. primary school), having an interest in technology, perceptions regarding developments in artificial intelligence, and the belief that AI has an emotional impact (*p* < 0.05). For the negative attitude subscale, only perceptions regarding developments in artificial intelligence were found to be significantly associated (*p* < 0.05).

**Conclusion:**

Medical students demonstrated generally positive attitudes toward AI. Higher paternal education, technological interest, perceiving AI as emotionally influential, and evaluating AI developments positively were predictors of favorable attitudes. In addition, more positive evaluations of AI-related developments were also associated with higher scores on the reverse-coded negative attitude subscale, indicating lower negative attitudes toward AI. These findings suggest that students’ attitudes toward AI are shaped not only by technological interest but also by perceptual factors related to AI. Therefore, integrating clinically oriented AI content and awareness-building activities into medical education may support the development of more balanced and informed attitudes toward AI.

**Supplementary Information:**

The online version contains supplementary material available at https://doi.org/10.1186/s12909-026-09859-4.

## Introduction

The concept of artificial intelligence (AI) was first introduced in 1955 through a scientific proposal presented at Dartmouth College. This proposal suggested that if every aspect of human intelligence could be precisely described, it could be simulated by machines, thereby laying the scientific foundations of AI research [1]. AI is commonly defined as computer systems capable of performing tasks that typically require human intelligence [2]. Such systems imitate various cognitive processes—including analyzing large datasets, drawing logical conclusions, generalizing information, and learning from experience [3].

As one of the fastest-growing technologies of the twenty-first century, AI has become a key component of digital transformation across multiple sectors, including economics, education, health, and medicine [4, 5]. In the medical field in particular, AI enables healthcare services to be delivered more efficiently, rapidly, and in a personalized manner [6, 7]. Early AI applications in medicine emerged with software designed to guide antimicrobial therapy; today, AI encompasses clinical decision-support systems, predictive analytics, and patient-management algorithms [8, 9]. These systems are widely used in routine care, public health surveillance, and epidemiological forecasting [5, 10]. AI has improved diagnostic accuracy in fields such as radiology, pathology, and dermatology, and several studies have shown that AI algorithms can achieve similar or even higher accuracy than expert clinicians in interpreting chest X-rays, classifying skin lesions, and conducting cancer screening [11].

Given these rapid developments, future physicians are increasingly expected to be familiar with machine learning, deep learning, and related techniques, as well as to understand the potential and limitations of these algorithms [12]. Therefore, the systematic integration of AI training into medical curricula has been widely recommended [13]. Although the global importance of AI education is well recognized [12, 14], research in this area remains limited, particularly in developing countries [15]. While many medical schools in high-income countries have already incorporated AI-related content into their curricula [16], the process is still in its early stages in developing regions [17]. The role of AI in medical education extends beyond technical knowledge, encompassing ethical, legal, and professional dimensions [18]. Indeed, the literature reports potential concerns associated with AI, such as weakened physician–patient relationships, job insecurity, and emotional stress during adaptation to technological change [19, 20]. Existing curricula also show major gaps in AI literacy, data ethics, and algorithmic bias [21]. Another study identified several challenges in integrating AI into medical education, including the absence of standards defining student AI literacy, insufficient awareness of proven clinical benefits, and a shortage of qualified instructors [22]. These issues highlight the need for evidence-based programs to integrate AI into the training of healthcare professionals [23].

AI is becoming increasingly prevalent in healthcare delivery and medical education; however, for these applications to be effectively aligned with clinical practice, they must be designed in close collaboration with clinicians and involve end-users at early stages [24, 25]. Previous studies emphasize that medical students—as future healthcare providers—will play a decisive role in the digital transformation of health systems through their awareness and attitudes toward AI [15, 26]. While several studies report that medical students tend to exhibit positive attitudes toward AI [15, 27], others indicate that concerns regarding ethical issues, professional uncertainty, and the potential reduction of the humanistic aspects of medicine may contribute to more cautious or negative perspectives [20, 28].

The expanding role of AI in medical education necessitates not only the development of technical competencies but also an understanding of how students perceive and adopt these technologies. Students’ positive or negative attitudes toward AI directly influence curriculum integration, acceptance, and ethical awareness. This study offers a unique contribution to the literature by evaluating medical students’ positive and negative attitudes toward AI as separate subdimensions and statistically examining their determinants. The findings are expected to provide essential baseline data for future program development, curriculum adaptation, and awareness-raising initiatives related to AI integration in medical education.

This study aims to assess medical students’ attitudes toward AI and identify the determinants of these attitudes. Additionally, it seeks to provide recommendations for educational strategies and awareness-raising approaches to support the integration of AI into medical education.

## Materials and methods

### Study design and participants

This descriptive cross-sectional study was conducted between January 22 and May 28, 2025, among sixth-year medical students enrolled at Pamukkale University Faculty of Medicine in Denizli, Türkiye. This group was selected because they were at the point of graduation, had clinical experience, and were close to beginning their professional medical careers.

### Sample size and sampling method

The study population consisted of all sixth-year medical students (*N* = 237). Since the population size was known, the minimum required sample size was calculated using OpenEpi with a 99.9% confidence level, ± 5% margin of error, and *p* = 0.50. The minimum sample size was determined to be 195. A census method was used, aiming to include the entire population. The study was completed with 198 students who voluntarily participated (response rate: 83.5%).

### Data collection tool

Data were collected using a Descriptive Information Form, which assessed students’ sociodemographic characteristics and their knowledge and experiences regarding AI, and the General Attitudes toward Artificial Intelligence Scale (GAAIS, Turkish version). Data were gathered face-to-face by the researcher in the classroom outside of course hours. All students were informed about the study, and the questionnaires took approximately 10–15 min to complete.

#### Descriptive information form

The dependent variables were the positive and negative attitude subscale scores of the General Attitudes toward Artificial Intelligence Scale. Independent variables included age, gender, parental education, family income, longest-lived place, interest in technology, general knowledge about AI, sources of information about AI, AI tools known, experience with AI in daily life, purposes of AI use, opinions about developments in AI, belief that AI changes work and daily life, and belief that AI affects them emotionally.

#### General attitudes toward artificial intelligence scale (GAAIS)

The scale was developed to measure individuals’ overall attitudes toward AI [29]. Its Turkish validity and reliability study was conducted by Kaya et al. The scale consists of 20 items and two subscales: Positive Attitude (12 items) and Negative Attitude (8 items). Items are rated on a 5-point Likert scale (1 = Strongly disagree, 5 = Strongly agree). Items in the negative attitude subscale are reverse-coded. Since both subscales are coded such that higher scores indicate more positive attitudes, they were evaluated independently rather than generating a total score, in accordance with the scale developer’s recommendation. Possible scores range from 12 to 60 for positive attitude and 8–40 for negative attitude. The Turkish version’s internal consistency (Cronbach’s alpha) was reported as 0.82 for positive attitude and 0.84 for negative attitude [30].

### Ethical considerations

Approval for the study was obtained from the Non-Interventional Clinical Research Ethics Committee of the university (dated January 21, 2025; No: E-60116787-020-643327), as well as institutional permission. Participation was voluntary, and written informed consent was obtained from all students.

### Statistical analysis

Data were analyzed using IBM SPSS Statistics 25.0. The normality of the data distribution was assessed using the Kolmogorov–Smirnov test and skewness–kurtosis values. Descriptive statistics were presented as frequencies, percentages, means ± standard deviations, and medians (minimum–maximum). Mann–Whitney U tests were used for two-group comparisons, and Kruskal–Wallis tests were used for comparisons involving more than two groups. When the Kruskal–Wallis test indicated a significant difference, Dunn–Bonferroni post-hoc tests were conducted.

Multiple linear regression analysis (backward method) was used to identify factors affecting positive and negative attitudes toward AI. Variables found to be significant in the bivariate analyses were included in the regression analyses. Statistical significance was set at *p* < 0.05.

## Results

The mean age of the participants was 24.47 ± 0.98 years, and 56.1% were female while 43.9% were male. Regarding parental education, 38.9% of the students’ mothers had a high school education, and 45.5% of their fathers had a university-level or higher education. A total of 59.1% of the students reported that their family income was equal to their expenses. Additionally, 72.2% of the students had lived primarily in a provincial city center (Table 1).

**Table 1:** Descriptive characteristics of the students (*n* = 198)

	Mean ± SD; Median (Min–Max)	*n*	%
Age (years)	24.47 ± 0.98; 24(23–27)		
Gender			
Male		87	43.9
﻿Female		111	56.1
Department			
Faculty of Medicine		198	100.0
Year of Study			
6th year		198	100.0
Mother’s education level			
Illiterate		4	2.0
Literate		9	4.5
Primary school graduate		22	11.1
Middle school graduate		18	9.1
High school graduate		77	38.9
University or higher		68	34.4
Father’s education level			
Illiterate		1	0.5
Literate		4	2.0
Primary school graduate		21	10.6
Middle school graduate		10	5.0
High school graduate		72	36.4
University or higher		90	45.5
Family income status			
Income less than expenses		23	11.6
Income equal to expenses		117	59.1
Income more than expenses		58	29.3
Place lived longest			
Province (city)		143	72.2
District		44	22.2
Village/Town		11	5.6

*SD* Standard Deviation, *Min* Minimum, *Max* Maximum

A total of 73.2% of the participants reported that developments in technology attracted their interest, and 74.2% stated that they had knowledge about AI. A total of 93.2% of the students reported social media as a source of information about AI. Regarding AI tools known, 100% of the students were aware of ChatGPT, while 29.9% reported knowing MidJourney, 24.5% Copilot, 21.8% Google Bard, and 21.1% DALL-E. In total, 76.3% of the participants reported having experience with AI in their daily lives. The most frequently reported purposes of use were completing assignments and conducting research at 76.8%, obtaining information at 61.6%, and curiosity at 53.6%. Regarding views on AI-related developments, 63.1% of the students considered these advancements positive. Additionally, 69.7% believed that AI has changed work and daily life, while 52.0% stated that AI did not affect them emotionally (Table 2).

**Table 2:** Students’ knowledge, experience, and opinions regarding artificial intelligence (*n* = 198)

		*n*	%
Interest in technological developments			
Yes		145	73.2
No		53	26.8
General knowledge about AI			
Yes		147	74.2
No		51	25.8
Sources of information about AI^a^ (*n* = 147)			
Scientific publications	Yes	58	39.5
	No	89	60.5
Seminars and conferences	Yes	41	27.9
	No	106	72.1
Social media	Yes	137	93.2
	No	10	6.8
Friends/peer group	Yes	3	2.0
	No	144	98.0
AI tools known^a^ (*n* = 147)			
ChatGPT	Yes	147	100.0
	No	0	0.0
DALL-E	Yes	31	21.1
	No	116	78.9
Copilot (GitHub Copilot)	Yes	36	24.5
	No	111	75.5
MidJourney	Yes	44	29.9
	No	103	70.1
Google Bard	Yes	32	21.8
	No	115	78.2
DeepSeek	Yes	8	5.4
	No	139	94.6
Grok	Yes	2	1.4
	No	145	98.6
Praktika	Yes	1	0.7
	No	146	99.3
Experience with AI in daily life			
Yes		151	76.3
No		47	23.7
Purpose of AI use^a^ (*n* = 151)			
Gaming	Yes	38	25.2
	No	113	74.8
Translation	Yes	74	49.0
	No	77	51.0
Assignments/Research	Yes	116	76.8
	No	35	23.2
Design	Yes	50	33.1
	No	101	66.9
Information seeking	Yes	93	61.6
	No	58	38.4
Curiosity	Yes	81	53.6
	No	70	46.4
Opinions about developments in AI			
Positive		125	63.1
Negative		14	7.1
Undecided		39	19.7
No opinion		20	10.1
Belief that AI changes work and daily life			
Yes		138	69.7
No		9	4.5
Partly		33	16.7
No opinion		18	9.1
Belief that AI affects them emotionally			
Yes		31	15.7
No		103	52.0
Partly		37	18.7
No opinion		27	13.6

^a^Multiple responses allowed

In the present study, the internal consistency coefficients (Cronbach’s alpha) were 0.93 for the positive attitude subscale and 0.87 for the negative attitude subscale. The mean score of the positive attitude subscale was 45.01 ± 9.17, while the mean score of the negative attitude subscale was 26.41 ± 6.22. There was no significant difference between gender and either the positive or negative attitude subscale scores (*p* > 0.05). A significant association was found between paternal education level and positive attitude scores (*p* = 0.030); however, post-hoc analysis revealed no significant pairwise differences between the groups.

A significant association was identified between family income status and negative attitude scores (*p* = 0.029). According to post-hoc analysis, the difference was observed between the groups reporting “income equal to expenses” and “income higher than expenses” (Adj. *p* < 0.05) (Table 3).

**Table 3:** Comparison of general attitudes toward AI scale scores by socio-demographic characteristics

	Positive		Negative	
	Mean ± SD; Median (Min–Max)	Z/H; *p*	Mean ± SD; Median (Min–Max)	Z/H; *p*
General Attitudes Toward AI Scale	45.01 ± 9.17; 46(12–60)	-	26.41 ± 6.22; 27(8–40)	-
Gender				
Male	46.00 ± 9.13; 47(12–60)	-1.898; 0.058	26.05 ± 6.61; 27(8–40)	-0.010; 0.992
Female	44.23 ± 9.17; 44(12–60)		26.69 ± 5.91; 27(14–40)	
Mother’s education level				
Illiterate	48.00 ± 11.34; 50(35–58)	0.339; 0.997	24.00 ± 7.16; 24(17–32)	4.760; 0.446
Literate	44.78 ± 8.64; 44(30–57)		26.78 ± 6.87; 26 (15–39)	
Primary school graduate	43.77 ± 11.17; 46(12–60)		28.45 ± 6.59; 28(14–40)	
Middle school graduate	44.89 ± 10.01; 46(12–60)		25.72 ± 5.77; 26(14–36)	
High school graduate	45.04 ± 8.81; 46(22–60)		26.95 ± 6.30; 27(8–40)	
University or higher	45.25 ± 8.89; 45(24–60)		25.41 ± 5.96; 26(12–39)	
Father’s education level				
Illiterate	58.00 ± 0; 58(58–58)	12.375; **0.030**	28.00 ± 0; 28(28–28)	7.124; 0.212
Literate	50.50 ± 7.19; 52(41–57)		31.00 ± 5.60; 30(26–39)	
Primary school graduate	40.62 ± 9.47; 41(24–57)		24.10 ± 5.20; 24(14–32)	
Middle school graduate	48.60 ± 8.28; 49(35–60)		29.40 ± 5.66; 28(22–40)	
High school graduate	43.60 ± 9.47; 45(12–60)		26.72 ± 6.83; 27(8–40)	
University or higher	46.37 ± 8.55; 46(24–60)		26.14 ± 5.88; 27(12–40)	
Family income status				
Income less than expenses	43.00 ± 13.16; 46(12–58)	0.564; 0.754	27.48 ± 6.96; 28(14–40)	7.092; **0.029**
Income equal to expenses	44.94 ± 8.34; 45(22–60)		27.00 ± 5.90; 27(8–40)^a^	
Income more than expenses	45.93 ± 8.95; 47(28–60)		24.79 ± 6.35; 25(12–40)^a^	
Place lived longest				
Province (city)	45.41 ± 9.17; 46(12–60)	4.073; 0.130	26.58 ± 6.28; 27(8–40)	0.406; 0.816
District	43.07 ± 8.92; 44(12–60)		26.16 ± 6.18; 26(14–40)	
Village/Town	47.45 ± 9.80; 48(24–58)		25.18 ± 5.95; 26(14–33)	

*Z* Mann-Whitney U, *H* Kruskal-Wallis, *SD* Standard deviation, *Min* Minimum, *Max* Maximum, ^a^Pairwise group difference

Participants who reported being interested in technological developments had significantly higher positive and negative attitude scores compared with those who were not interested (*p* < 0.05). Students who stated that they had general knowledge about AI also had significantly higher positive and negative attitude scores than those without such knowledge (*p* < 0.05).

Regarding sources of information, students who identified scientific publications as a source had significantly higher positive and negative attitude scores than those who did not (*p* < 0.05). Positive attitude scores were also significantly higher among students who reported seminars and conferences as an information source compared with those who did not (*p* = 0.002).

Students familiar with tools such as DALL-E (*p* = 0.001), Copilot (*p* = 0.004), MidJourney (*p* = 0.007), and Google Bard (*p* < 0.001) had significantly higher positive attitude scores toward AI compared with students unfamiliar with these tools. Moreover, students who knew MidJourney had significantly higher negative attitude scores than those who did not (*p* = 0.038).

Students who used AI in their daily lives also had significantly higher positive attitude scores than those who did not (*p* = 0.001). Positive attitude scores were significantly higher among students who used AI for assignments/research, design, information seeking, and curiosity compared with those who did not (*p* < 0.05).

A significant association was found between students’ perspectives on AI-related developments and their positive attitude scores (*p* < 0.001). According to post-hoc analysis, differences were observed between the positive–negative, positive–undecided, and positive–no opinion groups (Adj. *p* < 0.05). A significant association was also found between these perspectives and negative attitude scores (*p* < 0.001). Post-hoc analysis indicated differences between the negative–positive, negative–undecided, negative–no opinion, and positive–undecided groups (Adj. *p* < 0.05).

A significant relationship was detected between the belief that AI has changed work and daily life and positive attitude scores (*p* < 0.001). Post-hoc analysis indicated a difference between the “yes” and “no opinion” groups (Adj. *p* < 0.05).

Additionally, a significant association was found between students’ perceptions of the emotional impact of AI and their positive attitude scores (*p* = 0.002). Post-hoc analysis revealed differences between the yes–no, yes–partially, and yes–no opinion groups (Adj. *p* < 0.05) (Table 4).

**Table 4:** Comparison of AI attitude subscale scores by students’ AI-related knowledge, experience, and perceptions

		Positive		Negative	
		Mean ± SD; Median (Min–Max)	Z/H; *p*	Mean ± SD; Median (Min–Max)	Z/H; *p*
Interest in technological developments					
Yes		47.42 ± 7.50; 47(25–60)	-5.711; **<0.001**	27.06 ± 6.00; 27(8–40)	-2.668; **0.008**
No		38.40 ± 10.11; 39(12–60)		24.64 ± 6.52; 24(10–40)	
General knowledge about AI					
Yes		47.00 ± 7.84; 46(25–60)	-4.293; **<0.001**	26.99 ± 5.97; 27(8–40)	-2.099; **0.036**
No		39.25 ± 10.33; 42(12–57)		24.75 ± 6.67; 25(10–40)	
Sources of information about ^a^(*n* = 147)					
Scientific publications	Yes	50.05 ± 7.96; 49(25–60)	-4.175; **<0.001**	28.45 ± 6.92; 28(8–40)	-2.665; **0.008**
	No	45.01 ± 7.12; 44(28–60)		26.03 ± 5.09; 26(15–40)	
Seminars and conferences	Yes	49.76 ± 7.64; 49(25–60)	-3.099; **0.002**	28.10 ± 7.02; 29(8–40)	-1.890; 0.059
	No	45.93 ± 7.68; 45(28–60)		26.56 ± 5.49; 27(12–40)	
Social media	Yes	46.92 ± 7.98; 46(25–60)	-0.358; 0.720	26.93 ± 5.91; 27(8–40)	-0.979; 0.327
	No	48.10 ± 5.74; 46(42–58)		27.70 ± 7.09; 30(12–38)	
Friends/peer group	Yes	46.67 ± 12.22; 44(36–60)	-0.220; 0.826	29.67 ± 6.03; 29(24–36)	-0.721; 0.471
	No	47.01 ± 7.79; 46(25–60)		26.93 ± 5.98; 27(8–40)	
AI tools known ^a^(*n* = 147)					
ChatGPT	Yes	47.00 ± 7.84; 46(25–60)	-	26.99 ± 5.97; 27(8–40)	-
	No	—		—	
DALL-E	Yes	50.61 ± 8.70; 49(25–60)	-3.317; **0.001**	27.97 ± 6.46; 28(8–39)	-1.628; 0.104
	No	46.03 ± 7.33; 45(28–60)		26.72 ± 5.84; 27(12–40)	
Copilot (GitHub Copilot)	Yes	50.14 ± 8.14; 49(25–60)	-2.858; **0.004**	26.64 ± 6.40; 28(8–39)	-0.235; 0.814
	No	45.98 ± 7.49; 46(28–60)		27.10 ± 5.85; 27(12–40)	
MidJourney	Yes	49.43 ± 8.77; 48(25–60)	-2.707; **0.007**	28.30 ± 7.13; 28(8–40)	-2.071; **0.038**
	No	45.96 ± 7.20; 46(28–60)		26.43 ± 5.35; 26(12–40)	
Google Bard	Yes	50.87 ± 8.04; 52(25–60)	-3.509; **<0.001**	27.87 ± 5.27; 28(8–39)	-1.355; 0.175
	No	45.92 ± 7.46; 45(28–60)		26.74 ± 6.15; 27(12–40)	
DeepSeek	Yes	50.25 ± 6.96; 49(41–60)	-1.278; 0.201	27.75 ± 3.24; 28(24–32)	-0.496; 0.620
	No	46.81 ± 7.87; 46(25–60)		26.94 ± 6.10; 27(8–40)	
Grok	Yes	49.50 ± 6.36; 50(45–54)	-0.511; 0.610	28.50 ± 2.12; 29(27–30)	-0.545; 0.586
	No	46.97 ± 7.87; 46(25–60)		26.97 ± 6.01; 27(8–40)	
Praktika	Yes	45.00 ± 0; 45(45–45)	-0.283; 0.777	27.00 ± 0; 27(27–27)	0.000; 1.000
	No	47.01 ± 7.86; 46(25–60)		26.99 ± 5.99; 27(8–40)	
Experience with AI in daily life					
Yes		46.37 ± 8.45; 46(12–60)	-3.436; **0.001**	26.80 ± 5.94; 27(8–40)	-1.628; 0.103
No		40.62 ± 10.08; 43(12–60)		25.15 ± 6.97; 25(10–40)	
Purpose of AI use^a^					
Gaming	Yes	47.16 ± 10.58; 49(12–60)	-1.003; 0.316	26.71 ± 6.00; 28(12–39)	-0.606; 0.545
	No	46.11 ± 7.65; 46(24–60)		26.83 ± 5.94; 27(8–40)	
Translation	Yes	46.77 ± 8.75; 47(12–60)	-0.827; 0.408	26.45 ± 5.30; 26(14–39)	-1.162; 0.245
	No	45.99 ± 8.20; 46(24–60)		27.14 ± 6.51; 27(8–40)	
Assignments/Research	Yes	47.44 ± 8.19; 47(12–60)	-2.735; **0.006**	27.15 ± 5.72; 27(12–40)	-0.773; 0.439
	No	42.83 ± 8.46; 44(24–60)		25.66 ± 6.56; 27(8–38)	
Design	Yes	49.86 ± 6.41; 49(37–60)	-3.755; **<0.001**	27.36 ± 5.35; 28(15–39)	-0.959; 0.338
	No	44.64 ± 8.83; 44(12–60)		26.52 ± 6.22; 27(8–40)	
Information seeking	Yes	48.10 ± 8.17; 48(24–60)	-3.019; **0.003**	27.56 ± 5.56; 28(12–40)	-1.883; 0.060
	No	43.60 ± 8.22; 45(12–58)		25.59 ± 6.36; 26(8–40)	
Curiosity	Yes	47.93 ± 8.37; 48(25–60)	-2.309; **0.021**	27.21 ± 6.41; 27(8–40)	-1.055; 0.292
	No	44.57 ± 8.24; 45(12–60)		26.33 ± 5.35; 27(12–40)	
Opinions about developments in AI					
Positive		48.70 ± 7.26; 48(24–60)	58.855; **<0.001**	28.22 ± 5.54; 28(12–40)	36.843; **<0.001**
Negative		32.07 ± 10.81; 31(12–52)		18.00 ± 5.19; 18(10–28)	
Undecided		41.54 ± 5.69; 42(25–53)		24.15 ± 5.07; 24(8–34)	
No opinion		37.70 ± 9.47; 39(12–49)		25.35 ± 6.78; 25(15–40)	
Belief that AI changes work and daily life					
Yes		46.99 ± 8.38; 47(12–60)	25.088; **<0.001**	26.91 ± 6.15; 27(12–40)	7.512; 0.057
No		38.56 ± 12.26; 42(22–57)		21.22 ± 5.93; 24(10–28)	
Partly		43.36 ± 7.27; 44(25–60)		26.42 ± 5.76; 27(8–40)	
No opinion		36.00 ± 9.65; 37(12–49)		25.11 ± 6.83; 24(14–40)	
Belief that AI affects them emotionally					
Yes		50.13 ± 8.01; 48(24–60)	14.583; **0.002**	28.87 ± 7.95; 28(12–40)	4.925; 0.177
No		44.38 ± 9.02; 45(12–60)		25.77 ± 5.59; 26(10–40)	
Partly		44.68 ± 8.37; 44(24–60)		25.92 ± 6.12; 26(8–35)	
No opinion		41.96 ± 10.23; 44(12–58)		26.70 ± 6.04; 27(15–40)	

Values in bold indicate statistical significance (*p* <0.05)

*Z* Mann- Whitney U, *H* Kruskal-Wallis

^a^Multiple responses allowed

It was determined that a one-unit increase in paternal education level increased the positive attitude score by 3.157 points (β = 0.172; *p* = 0.002). A one-unit increase in interest in technology was associated with a 5.24-point increase in positive attitude scores (β = 0.254; *p* < 0.001).

AI-related developments positively increased the positive attitude score by 14.102 points (β = 0.744; *p* < 0.001). Similarly, A one-unit increase in being undecided about AI increased the positive attitude score by 8.485 points (β = 0.369; *p* < 0.001). A one-unit increase in reporting having no opinion about AI increased the positive attitude score by 6.491 points (β = 0.214; *p* = 0.008).

Among students who believed that AI could have an emotional impact, a one-unit increase in this perception increased the positive attitude score by 3.980 points (β = 0.158; *p* = 0.004) (Table 5).

**Table 5:** Factors associated with the positive subscale of the general attitudes toward artificial intelligence scale

Variables	B	Std. Error	β	95% CI for B	t	*p*
Father’s educational level *Primary school (Reference)*	**-**	**-**	**-**	**-**	**-**	**-**
University	3.157	1.028	0.172	1.128–5.185	3.070	**0.002**
Interest in technology No *(Reference)*	-	-	-	-	-	**-**
Yes	5.242	1.201	0.254	2.873–7.610	4.365	**< 0.001**
Opinions about developments in AI *Negative (Reference)*	-	-	-	-	-	**-**
Positive	14.102	2.042	0.744	10.074–18.129	6.907	**< 0.001**
Undecided	8.485	2.197	0.369	4.151–12.818	3.862	**< 0.001**
No opinion	6.491	2.433	0.214	1.692–11.291	2.668	**0.008**
Belief that AI affects them emotionally *No opinion (Reference)*						
Yes	3.980	1.378	0.158	1.261–6.699	2.887	**0.004 **

Values in bold indicate statistical significance (*p* < 0.05)

A multiple linear regression analysis (backward method) was conducted. No multicollinearity was detected among the independent variables (VIF = 1.04–4.03). R² = 0.456 / Adjusted R² = 0.433

Variables included in the model: age, gender, father’s education level, interest in technology, general knowledge about AI, experience with AI in daily life, opinions about developments in AI, belief that AI changes work and daily life, and belief that AI affects them emotionally

It was found that a one-unit increase in the level of evaluating AI-related developments positively increased the negative attitude score by 9.847 points (β = 0.766; *p* < 0.001). A one-unit increase in indecision regarding AI increased the negative attitude score by 5.751 points (β = 0.369; *p* = 0.001). Additionally, a one-unit increase in reporting having no opinion about AI increased the negative attitude score by 7.102 points (β = 0.345; *p* < 0.001) (Table 6).

**Table 6:** Factors associated with the negative subscale of the general attitudes toward artificial intelligence scale

Variables	B	Std. Error	β	95% CI for B	t	*p*
Opinions about developments in AI *Negative (Reference)*	**-**	**-**	**-**	**-**	**-**	**-**
Positive	9.847	1.569	0.766	6.751–12.942	6.274	**< 0.001**
Undecided	5.751	1.734	0.369	2.331–9.171	3.316	**0.001**
No opinion	7.102	1.929	0.345	3.296–10.907	3.681	**< 0.001**

Values in bold indicate statistical significance (*p* < 0.05)

A multiple linear regression analysis (backward method) was performed. No multicollinearity was detected among the independent variables (VIF = 2.19–3.72). R² = 0.227 / Adjusted R² = 0.211

Variables included in the model: age, gender, family income status, interest in technology, general knowledge about AI, opinions about developments in AI, and belief that AI changes work and daily life

## Discussion

### Evaluation of general attitude levels toward artificial intelligence

In this study, the mean positive attitude score of the students was 45.01 ± 9.17. In Türkiye, a study among medical and dental students reported a similar mean positive attitude score of 42.83 ± 0.59 [31]. Likewise, a study conducted among nurses found a positive attitude score of 43.74 ± 6.87 [32].

International studies also demonstrate predominantly positive perceptions of AI. For example, research conducted in Pakistan among physicians reported that participants generally adopted a favorable stance toward AI [33]. In a study from the United Kingdom, the majority of medical students believed that AI would play a significant role in future healthcare and considered receiving AI education beneficial for their careers [34]. Similarly, a study involving medical students and physicians from the UK and Middle Eastern countries reported that most participants believed AI would be important in modern medicine, suggesting that AI could reduce diagnostic errors and serve as a supportive tool for clinicians [35]. These findings are consistent with the generally positive attitude trends observed in our study. Collectively, previous studies suggest that many medical students and healthcare trainees perceive AI as a potentially useful and supportive component of future healthcare practice.

In the present study, the mean negative attitude score was 26.41 ± 6.22. Comparable studies in Türkiye reported a negative attitude score of 24.57 ± 0.40 among medical and dental students [31], and 25.53 ± 5.64 among nurses [32]. Similarly, a study among physicians in Pakistan found low levels of negative attitudes, indicating limited concerns regarding AI [33]. However, findings from Sweden revealed that one-third of medical students were fearful of the increasing use of AI and stated that such concerns could influence their specialty preferences [36]. Another study reported that nearly half of the students feared AI could create trust issues in the physician–patient relationship and diminish professional value [37]. Additional evidence suggests that ethical ambiguities and fears of losing control may contribute to cautious or negative attitudes among students [38].

Overall, the results indicate that while positive attitudes toward AI are more prominent, negative attitudes are not negligible. In this context, medical education programs should address not only the opportunities offered by AI but also its ethical, legal, and professional dimensions, which may be considered underlying concerns. In this regard, integrating structured case-based discussions into curricula may enable students to critically evaluate issues such as algorithmic bias, data privacy, accountability, and the physician–patient relationship through realistic scenarios in AI-supported clinical decision-making processes.

Furthermore, incorporating these topics into medical ethics courses and clinical clerkships may support students in evaluating the role of AI in clinical practice alongside their professional responsibilities. In addition, to balance students’ cautious or negative perceptions of AI, its opportunities and limitations should be addressed in a comprehensive manner through short modules, elective courses, and simulation-based applications.

### Determinants of positive attitudes toward artificial intelligence

In this study, students whose fathers had a university-level education exhibited significantly higher positive attitude scores toward AI compared with those whose fathers had only primary school education. Similarly, previous research has shown that children from families with higher educational backgrounds tend to have advantages in both digital skills and positive attitudes toward technology [39]. In contrast to our findings, a study among university students reported that maternal, rather than paternal, education level was significantly associated with AI attitudes [40]. Another study investigating attitudes toward technology found that attitudes were related to mothers’ educational levels but not to fathers’ [41]. These discrepancies in the literature may reflect the traditionally stronger caregiving and educational roles attributed to mothers during childhood development. Nevertheless, existing studies suggest that parental education—regardless of whether maternal or paternal—may play a meaningful role in shaping attitudes toward technology and AI.

Although the present study was not designed based on theoretical frameworks, theories in health professions education research can be valuable not only for guiding study design but also for enhancing the interpretive lens of data analysis and discussion [42]. In this context, the influence of parental educational background on students’ digital competencies and technology-related perceptions may be interpreted through the lens of cultural capital theory. Prior research indicates that children from families with higher cultural capital demonstrate more advanced digital skills and tend to use the internet more efficiently and for educational purposes [43]. Students raised in highly educated families may be more exposed to technological innovations and more likely to recognize the potential benefits of technology in both daily life and professional practice [44]. Conversely, another study found that socio-culturally disadvantaged students developed significantly more positive attitudes toward AI after receiving AI-focused training [45]. In this context, integrating AI-related educational content into medical curricula—particularly through case-based, simulation-supported, and clinically contextualized learning environments—may help mitigate disparities related to family background and enhance perceived usefulness and technology acceptance among students [45, 46].

At this point, introductory-level modules that include basic AI concepts and clinical applications can be developed, particularly for students from families with lower socio-cultural and educational backgrounds. These modules can be delivered through clinical scenarios involving the role of artificial intelligence in diagnostic support systems, risk prediction, and patient management, thereby enabling students to gain practical experience.

In addition, students’ perceptions of AI, levels of trust, and expectations regarding their professional roles can be addressed in a structured manner through simulation-based applications and small-group discussions. Such structured educational approaches may help reduce digital inequalities among students from different family and educational backgrounds and support the development of more balanced attitudes.

In this study, students who expressed interest in technological developments had significantly higher positive attitude scores compared with those who were not interested. Interestingly, Bewersdorff et al. reported the reverse directionality of this relationship, showing that positive attitudes toward AI increased students’ interest in AI, and this relationship was supported through a path model [47]. Taken together, these findings suggest a reciprocal relationship between interest in technology and attitudes toward AI.

Moreover, the present findings align with the Technology Acceptance Model (TAM), particularly its emphasis on perceived usefulness. According to TAM, individuals’ attitudes toward a technology are shaped by the extent to which they perceive the technology as easy to use, manageable, and requiring minimal effort [48, 49]. In the extended Unified Theory of Acceptance and Use of Technology 2 (UTAUT2), effort expectancy and hedonic motivation are also noted to strengthen positive attitudes and behavioral intentions toward technology use [49].

Students with a strong interest in technology may perceive AI applications as more understandable, less complex, and more accessible. As a result, they may view AI not as a complex system but as an adaptable and supportive tool in clinical practice. A growing interest in technology can reinforce the perceived ease of use, thereby contributing to higher positive attitude scores. Therefore, in medical education, AI-related educational content should be structured in a practice-based, experience-oriented manner that provides step-by-step learning opportunities. In this regard, progressive learning modules covering fundamental AI concepts and their use can be developed, particularly for students with low interest in technology. These modules can be designed in a simple-to-complex sequence, enabling students to experience AI-supported clinical applications (e.g., diagnostic support systems and risk prediction) in low-risk learning environments.

In addition, encouraging students to actively use AI tools through simulation-based applications and small-group activities may enhance perceived ease of use. Furthermore, short hands-on sessions and guided practice offered at early stages may increase engagement with AI among students with low interest in technology, while reducing perceptions of anxiety and complexity. Such structured and progressive educational approaches may support the development of positive attitudes toward AI by strengthening perceived ease of use.

In this study, having knowledge and experience with AI increased positive attitude scores in bivariate analyses, but the association did not remain significant in the multiple regression model. Similar observations have been reported in the literature. For example, one study found a significant bivariate relationship between AI experience and attitudes, but this direct effect diminished in regression models when mediating factors such as perceived usefulness were included [50]. In a national-level study, Gnambs et al. reported that although experience correlated with attitudes, experience alone was not a strong predictor in multivariable analyses [51]. These patterns align with TAM, which posits that knowledge and experience are not direct determinants of attitudes; instead, they function as antecedent variables influencing perceived usefulness and perceived ease of use [48, 49].Therefore, in medical education, merely increasing the level of knowledge is not sufficient. In this regard, structured educational approaches should be developed to enable students to directly experience the clinical benefits of AI. For this purpose, scenario-based applications integrated into the curriculum may allow students to experience AI-supported diagnosis, risk prediction, and clinical decision-making processes.

In addition, small-group case discussions may encourage students to critically evaluate the strengths and limitations of AI. Simulation-based applications and guided practice sessions may further enhance perceived ease of use by enabling students to actively engage with AI tools. Such structured and experience-oriented educational strategies may go beyond simple knowledge transfer by strengthening key determinants such as perceived usefulness and ease of use, thereby more effectively supporting the development of positive attitudes toward AI.

The present study also found that students who evaluated AI-related developments positively had significantly higher positive attitude scores than those who viewed them negatively. This finding is consistent with research demonstrating that positive perceptions of AI technologies and openness to innovation strengthen attitudes and acceptance levels. For example, Serdenia et al. reported that among teacher candidates, positive perceptions of AI effectiveness significantly increased attitudes and acceptance levels [52]. Similarly, Granström and Oppi found that teachers’ perceptions of AI usefulness and efficiency were strongly associated with positive attitudes and higher adoption levels [53]. Within the TAM framework, these findings can be explained by perceived usefulness. The belief that a technology enhances professional performance is considered a key determinant of positive attitudes toward that technology [48, 49]. Therefore, students who perceive AI as potentially beneficial for clinical decision-making, diagnostic accuracy, or workload reduction may be more likely to report positive attitudes.

Interestingly, students who selected “undecided” or “no opinion” also had significantly higher positive attitude scores compared with those expressing negative views. This suggests that even when students lack a firm position, they tend to lean toward a more positive perception of AI. Similarly, Longoni et al. noted that cautious attitudes toward medical AI do not necessarily reflect rejection but may stem from curiosity and expectations regarding its potential contributions to healthcare [26]. Therefore, in medical education, it is important to increase learning experiences that make the concrete clinical contributions of artificial intelligence (AI) more visible. In this regard, integrating real clinical case examples into the curriculum may enable students to analyze AI-supported diagnostic and treatment processes. In particular, structured case discussions in which AI outputs are incorporated into clinical decision-making may allow students to directly observe the clinical benefits of the technology.

In addition, simulation-based applications may provide opportunities for students to experience AI-supported scenarios in low-risk environments. Furthermore, short hands-on sessions and guided analysis activities may encourage students to actively evaluate the contributions of AI. Such experience-oriented educational approaches may strengthen perceived usefulness and thereby contribute to more positive attitudes, particularly among students who are undecided or hold neutral views toward AI.

In this study, students who believed that AI had an emotional impact exhibited significantly higher positive attitude scores. Kandemir et al. similarly reported that emotions toward AI were a significant determinant of attitudes among nurses [32]. International studies also show that emotional responses to AI—such as anxiety or excitement—significantly influence nursing students’ and healthcare trainees’ attitudes toward technology [54]. Another study emphasized that emotional reactions to AI in healthcare—such as trust, hope, or concern—play a crucial role in shaping attitudes among students and physicians [55]. Likewise, research has demonstrated that emotional perceptions toward AI strongly affect technology acceptance in health services [26]. This finding is also compatible with TAM3, which identifies emotional constructs such as “computer anxiety” and “perceived enjoyment” as strong determinants of attitudes [56]. In this context, it can be suggested that students who believe that artificial intelligence (AI) has an emotional impact on individuals tend to establish a more positive relationship with this technology. Positive emotional perceptions toward technology may be associated with viewing AI as less threatening and more supportive. This finding indicates that, in addition to cognitive evaluations such as perceived usefulness and ease of use, emotional perceptions may also play an important role in the process of technology adoption.

Accordingly, the finding that students who perceive AI as having an emotional impact exhibit higher positive attitude scores in our study is consistent with the literature emphasizing the importance of emotional factors in the technology acceptance process [26, 49].

This finding indicates that, in medical education, not only the transfer of knowledge but also learning environments that foster emotional awareness toward artificial intelligence (AI) are important. In this regard, structured reflection sessions in which students can express their feelings—such as anxiety, curiosity, and trust—regarding AI can be integrated into the curriculum.

In addition, through ethics- and communication-focused small-group discussions, the impact of AI on the physician–patient relationship can be addressed from a human-centered care perspective. Scenario-based applications that make the role of AI in clinical case examples more visible may also support students in developing a more positive emotional connection with this technology. Such holistic and experience-oriented educational approaches may contribute to the development of more positive overall attitudes toward AI by strengthening emotional perceptions.

### Determinants of negative attitudes toward artificial intelligence

In this study, students who held positive views about AI-related developments had significantly higher negative attitude scores compared with those who held negative views. Likewise, students who described themselves as undecided or having no opinion also showed significantly increased negative attitude scores. Given that the negative subscale of the instrument is reverse-coded, these findings indicate that students who view AI developments positively, or who express indecision or lack of opinion, actually exhibit more favorable attitudes toward AI than those who hold negative views.

Although AI knowledge appeared to be associated with negative attitudes in the bivariate analyses, this relationship became non-significant in the multiple regression model. Notably, negative attitude scores were predicted only by students’ perceptions of AI-related developments. This pattern suggests that students tend to interpret AI not merely as a technical tool but within the broader context of professional identity and clinical roles, implying that their attitudes are shaped more by perceptions and beliefs than by knowledge level alone [24]. Therefore, in medical education, beyond the delivery of theoretical knowledge, it is essential to employ scenario-based clinical applications, ethical deliberation sessions, and case discussions that clarify role boundaries, enabling students to reframe their perceptions of AI [24]. Thus, rather than focusing solely on the functional features of AI, medical curricula should include reflective activities addressing cognitive evaluations related to the future of the medical profession, decision-making autonomy, and role transformation [57].

These findings also suggest that attitudes toward AI are not unidimensional but instead reflect a bipolar or ambivalent structure. According to the Ambivalence Model, individuals can simultaneously hold both positive and negative evaluations of a technology; however, the dominant emotional evaluation ultimately guides the overall direction of their attitudes [58]. In this study, the increased negative attitude scores among students who responded “undecided” or “no opinion”—due to reverse coding—indicate a shift away from negative evaluations toward a more neutral or positive stance. This implies that these students may not reject AI outright but instead express cautious acceptance or measured optimism. In other words, they may recognize the potential benefits of AI while remaining cautious due to uncertainties associated with its implementation. Previous studies similarly report that attitudes toward AI in healthcare often oscillate between expectations of utility (e.g., reducing errors, improving efficiency) and concerns about loss of control or ethical ambiguity, thereby reinforcing the ambivalent nature of these attitudes [24, 26].

In medical education, it should be acknowledged that both positive and negative perceptions of artificial intelligence (AI) may coexist. In this regard, structured educational approaches integrated into the curriculum may help students address these ambivalent attitudes. In particular, scenario-based case discussions that make the role of AI in clinical decision-making visible may encourage students to evaluate both the benefits and limitations of the technology simultaneously.

In addition, reflection sessions addressing topics such as professional identity, physician autonomy, and ethical responsibilities may deepen students’ cognitive and emotional evaluations of AI. Furthermore, simulation-based applications involving uncertainty management and decision-support processes may enable students to experience interactions with AI in safe learning environments. Such holistic and structured educational approaches may help students to reconcile their ambivalent attitudes within a more balanced and positive framework and to position AI as a tool that supports their professional roles.

### Educational implications

The findings of this study provide several practical implications for the integration of artificial intelligence into medical education. Since interest in technology and positive perceptions of AI developments were associated with more favorable attitudes, medical curricula may benefit from introductory AI learning activities that demonstrate the clinical relevance, potential benefits, and limitations of AI in a balanced manner. In particular, such introductory content may be especially valuable for students with lower levels of technological interest or exposure. Rather than presenting AI only as a technical topic, educational content should include clinically contextualized examples, such as AI-supported diagnostic decision-making, risk prediction, and patient safety scenarios.

The finding that students’ perceptions of AI-related developments were associated with both positive and negative attitude subscale scores suggests that perception-oriented educational strategies may be particularly important. This dual pattern indicates that students may simultaneously recognize the benefits of AI while also experiencing uncertainty or concern. Therefore, structured case-based discussions may help students critically evaluate both the opportunities and risks of AI, including algorithmic bias, data privacy, accountability, and the physician’s role in AI-supported decision-making.

In addition, because emotional perceptions of AI were associated with more positive attitudes, AI education should include reflective components that allow students to discuss expectations, uncertainty, trust, anxiety, and professional identity. Such activities may help students process their emotional responses and develop a balanced understanding of AI without framing it either as a threat or as an unquestioned solution.

Based on these findings, the following practical checklist may support the integration of AI into medical education:

- Include introductory AI literacy sessions covering basic concepts, clinical uses, and limitations of AI, particularly for students with lower levels of technological interest, as interest in technology was associated with more favorable attitudes.
- Use case-based discussions to demonstrate how AI may support, but not replace, clinical reasoning, particularly given that students exhibited both positive and negative perceptions of AI developments.
- Incorporate ethical scenarios addressing bias, privacy, accountability, and patient–physician relationships to help students critically engage with identified concerns.
- Provide reflective sessions where students can discuss emotional responses, uncertainty, and professional role concerns related to AI, as emotional perceptions were associated with more positive attitudes.
- Ensure that AI learning opportunities are accessible to students with different levels of prior technological interest or exposure.
- Evaluate students’ AI-related attitudes and perceived readiness before and after educational interventions to monitor the effectiveness of training.

This checklist may serve as a practical framework for supporting the integration of AI into medical education.

### Limitations

This study has several limitations. First, the data were collected from sixth-year students at a single medical school, which may limit the generalizability of the findings to other institutions and student populations.

Second, due to the cross-sectional design, causal inferences cannot be established, and the directionality of the observed relationships remains uncertain. In addition, this design does not allow for the assessment of changes in attitudes over time.

Third, because the data were based on self-reported measures, participants’ responses may be subject to reporting bias and may not fully reflect their actual attitudes or behaviors.

Finally, the present study was not initially developed within a specific theoretical framework. Therefore, theoretical perspectives referenced in the literature (e.g., TAM, UTAUT2, the Ambivalence Model, and Cultural Capital Theory) were used retrospectively to aid the interpretation of the findings. Future studies may benefit from adopting a theory-informed approach to better understand the factors influencing medical students’ attitudes toward AI.

## Conclusions

This study found that medical students generally held positive attitudes toward AI, and these attitudes were significantly influenced by specific individual and perceptual factors. In particular, higher paternal education level, greater interest in technology, positive evaluations of AI-related developments, and the belief that AI can have an emotional impact were key variables that increased positive attitude scores. These findings may suggest that many students in the present sample tended to perceive AI as a supportive tool rather than a threat to clinical practice. Such positive tendencies indicate that awareness initiatives and technology-enhanced educational interventions related to AI may be well received among medical students.

Additionally, the finding that negative attitude scores were associated only with students’ perceptions of AI developments suggests that unfavorable attitudes are shaped by a narrow perceptual component rather than broad demographic or cognitive determinants. This may suggest that negative attitudes toward AI could potentially be addressed through targeted educational interventions. Accordingly, the results provide an important foundation for designing programs aimed at fostering AI awareness and competency in medical education.

In this context, integrating structured AI-related coursework and practical applications into the medical curriculum may support students in using these technologies consciously, ethically, and effectively. Given that attitudes are modifiable, these findings underscore the need for medical schools to systematically address AI-related content. Particularly during clinical years, implementing case-based AI applications, ethics and decision-making scenarios, and discussions about professional roles and competencies may strengthen students’ positive attitudes while reducing uncertainty- and distrust-based negative perceptions. Overall, the findings highlight the necessity of integrating AI-focused educational content into medical training programs.

## Supplementary Information

Supplementary Material 1.

## Data Availability

The datasets generated and/or analyzed during the current study are not publicly available due to participant confidentiality but are available from the corresponding author on reasonable request.
