# Supplementary material for: Determinants of medical students’ attitudes toward artificial intelligence: a cross-sectional study and implications for medical education
Source: BMC Med Educ. 2026 Jul 8;26:1554. doi: 10.1186/s12909-026-09859-4 (PMC13628730; doi:10.1186/s12909-026-09859-4)
Supplement: Supplementary file 1 [file 12909_2026_9859_MOESM1_ESM.pdf]

Supplementary File S1 — Socio-Demographic Questionnaire (English Version)

Section A — Socio-Demographic Information

1. Gender: 1 = Male ( ) 2 = Female ( )

2. Age: ______

3. Mother's education level:
1 = Illiterate ( ) 2 = Literate ( ) 3 = Primary school ( ) 4 = Middle school ( ) 5 = High school ( ) 6 = University or higher ( )

4. Father's education level:
1 = Illiterate ( ) 2 = Literate ( ) 3 = Primary school ( ) 4 = Middle school ( ) 5 = High school ( ) 6 = University or higher ( )

5. Household income status:
1 = Income is less than expenses ( ) 2 = Income equals expenses ( ) 3 = Income is more than expenses ( )

6. Do you smoke? 1 = Yes ( ) 2 = No ( )

7. Do you consume alcohol? 1 = Yes ( ) 2 = No ( )

8. Place where you lived the longest:
1 = City ( ) 2 = District ( ) 3 = Village/Town ( )

9. Are you interested in technological developments? 1 = Yes ( ) 2 = No ( )

10. Do you have general knowledge about artificial intelligence? 1 = Yes ( ) 2 = No ( ) (If No, skip to Question 13)

11. What is your source of information about artificial intelligence? (You may select more than one)
1 = Scientific publications ( ) 2 = Seminars/Conferences ( ) 3 = Social media ( ) 4 = Other: ____________

12. Which artificial intelligence tools do you know? (You may select more than one)
1 = ChatGPT ( ) 2 = DALL-E ( ) 3 = Copilot ( ) 4 = MidJourney ( ) 5 = Google Bard ( ) 6 = Other: ____________

13. Do you have experience using artificial intelligence in daily life?
(e.g., using an AI platform, application, or service)
1 = Yes ( ) 2 = No ( ) (If No, skip to Question 15)

14. If Yes, what are your purposes for using AI? (Select more than one)
1 = Gaming ( ) 2 = Translation ( ) 3 = Homework/Research ( ) 4 = Design ( ) 5 = Obtaining information ( ) 6 = Curiosity ( )

15. What is your opinion about developments related to artificial intelligence?
 1 = Positive ( ) 2 = Negative ( ) 3 = Undecided ( ) 4 = No opinion ( )

16. Do you think artificial intelligence is changing work life and daily life?
 1 = Yes ( ) 2 = No ( ) 3 = Partially ( ) 4 = No opinion ( )

17. Do you think artificial intelligence affects you emotionally?
 1 = Yes ( ) 2 = No ( ) 3 = Partially ( ) 4 = No opinion ( )

Section B — General Attitudes Toward Artificial Intelligence Scale (GAAIS)

The scale was developed to measure individuals' overall attitudes toward AI (Schepman & Rodway 2022).

Original English items, with item numbers matching the Turkish version under:

1. I am interested in using artificially intelligent systems in my daily life.
2. There are many beneficial applications of Artificial Intelligence.
3. Artificial Intelligence is exciting.
4. Artificial Intelligence can provide new economic opportunities for this country.
5. I would like to use Artificial Intelligence in my own job.
6. An artificially intelligent agent would be better than an employee in many routine jobs.
7. I am impressed by what Artificial Intelligence can do.
8. Artificial Intelligence can have positive impacts on people's wellbeing.
9. Artificially intelligent systems can help people feel happier.
10. Artificially intelligent systems can perform better than humans.
11. Much of society will benefit from a future full of Artificial Intelligence.
12. For routine transactions, I would rather interact with an artificially intelligent system than with a human.
13. I think Artificial Intelligence is dangerous.
14. Organisations use Artificial Intelligence unethically.
15. I find Artificial Intelligence sinister.
16. Artificial Intelligence is used to spy on people.

17. I shiver with discomfort when I think about future uses of Artificial Intelligence.
18. Artificial Intelligence might take control of people.
19. I think artificially intelligent systems make many errors.
20. People like me will suffer if Artificial Intelligence is used more and more.

Supplementary Material – Turkish Version of the General Attitudes to Artificial Intelligence Scale

Yapay Zekâya Yönelik Genel Tutum Ölçeği, Kaya *et al*. (2022).

Yönerge: Değerli katılımcı. Bu anket yapay zekaya yönelik tutumlarınız hakkında bilgi almak için tasarlanmıştır. Lütfen her bir maddeyi dikkatle okuyarak size en uygun olan seçeneği 1 ile 5 arasında işaretleyiniz.	Kesinlikle katılmıyorum	Katılmıyorum	Kararsızım	Katılıyorum	Kesinlikle katılıyorum
1. Günlük hayatımda yapay zekâ sistemlerini kullanmak ilgimi çekiyor.	1	2	3	4	5
2. Yapay zekânın birçok faydalı uygulaması vardır.	1	2	3	4	5
3. Yapay zekâ heyecan vericidir.	1	2	3	4	5
4. Yapay zekâ bu ülke için yeni ekonomik fırsatlar sağlayabilir.	1	2	3	4	5
5. Yapay zekâyı kendi işimde kullanmak isterim.	1	2	3	4	5
6. Yapay zekâya sahip bir yazılım/robot, birçok rutin işi bir insandan daha iyi yapabilir.	1	2	3	4	5
7. Yapay zekânın yapabileceklerinden etkilendim.	1	2	3	4	5
8. Yapay zekanın insanların iyi oluşları üzerinde olumlu etkileri olabilir.	1	2	3	4	5
9. Yapay zekâlı sistemler insanların daha mutlu hissetmelerine yardımcı olabilir.	1	2	3	4	5

10. Yapay zekâlı sistemler insanlardan daha iyi performans gösterebilir.	1	2	3	4	5
11. Toplumun çoğu, yapay zekâ ile donatılmış bir gelecekten faydalanacaktır.	1	2	3	4	5
12. Rutin işlemler için, bir insan yerine yapay zekâlı bir sistemle etkileşime girmeyi tercih ederim.	1	2	3	4	5
13. Yapay zekânın tehlikeli olduğunu düşünüyorum.	1	2	3	4	5
14. Kuruluşlar yapay zekâyı etik olmayan bir şekilde kullanırlar.	1	2	3	4	5
15. Yapay zekâyı şeytani/kötü niyetli buluyorum.	1	2	3	4	5
16. Yapay zekâ insanları gözetlemek için kullanılır.	1	2	3	4	5
17. Yapay zekânın gelecekteki kullanımlarını düşündüğümde üzüntüden titriyorum.	1	2	3	4	5
18. Yapay zekâ insanların kontrolünü ele geçirebilir.	1	2	3	4	5
19. Yapay zekâlı sistemlerin birçok hata yaptığını düşünüyorum.	1	2	3	4	5
20. Yapay zekâ gitgide daha fazla kullanılırsa benim gibi insanların zarar göreceğini düşünüyorum.	1	2	3	4	5

References

Kaya, F., Aydin, F., Schepman, A., Rodway, P., Yetişensoy, O., & Demir Kaya, M. (2022). The roles of personality traits, AI anxiety, and demographic factors in attitudes towards artificial intelligence. *International Journal of Human–Computer Interaction.* https://doi.org/10.1080/10447318.2022.2151730

Schepman, A. & Rodway, P. (2022). The General Attitudes towards Artificial Intelligence Scale (GAAIS): Confirmatory Validation and Associations with Personality, Corporate Distrust, and General Trust. *International Journal of Human–Computer Interaction* https://doi.org/10.1080/10447318.2022.2085400
